# Beyond Allergies—Updates on The Role of Mas-Related G-Protein-Coupled Receptor X2 in Chronic Urticaria and Atopic Dermatitis

**DOI:** 10.3390/cells13030220

**Published:** 2024-01-25

**Authors:** Liron Lerner, Magda Babina, Torsten Zuberbier, Katarina Stevanovic

**Affiliations:** 1 Institute of Allergology, Charité—Universitätsmedizin Berlin, 12203 Berlin, Germany; liron.lerner@charite.de (L.L.); magda.babina@charite.de (M.B.); torsten.zuberbier@charite.de (T.Z.); 2 Fraunhofer Institute for Translational Medicine and Pharmacology (ITMP), Allergology and Immunology, 12203 Berlin, Germany

**Keywords:** skin mast cells, MRGPRX2, urticaria, atopic dermatitis

## Abstract

Mast cells (MCs) are an important part of the immune system, responding both to pathogens and toxins, but they also play an important role in allergic diseases, where recent data show that non-IgE-mediated activation is also of relevance, especially in chronic urticaria (CU) and atopic dermatitis (AD). Skin MCs express Mas-related G-protein-coupled receptor X2 (MRGPRX2), a key protein in non-IgE-dependent MC degranulation, and its overactivity is one of the triggering factors for the above-mentioned diseases, making MRGPRX2 a potential therapeutic target. Reviewing the latest literature revealed our need to focus on the discovery of MRGPRX2 activators as well as the ongoing vast research towards finding specific MRGPRX2 inhibitors for potential therapeutic approaches. Most of these studies are in their preliminary stages, with one drug currently being investigated in a clinical trial. Future studies and improved model systems are needed to verify whether any of these inhibitors may have the potential to be the next therapeutic treatment for CU, AD, and other pseudo-allergic reactions.

## 1. Introduction

Inflammatory skin diseases have an adverse impact on the physical, psychological, and social wellbeing of patients, thereby highly compromising their quality of life along with that of their caregivers [1,2,3]. Atopic dermatitis (AD) and chronic urticaria (CU) are examples of such diseases, with an increasing global prevalence [4,5,6]. The major challenge in the treatment of such diseases is that they are complex and heterogeneous with respect to their pathogenesis. Nevertheless, the adverse impact of these chronic inflammatory skin diseases on public health highlights the need for improved treatment approaches and novel therapeutic agents [3,7,8]. Understanding the underlying pathophysiology of these skin diseases is essential for the discovery of novel therapeutic and preventative strategies.

The skin, the body’s largest organ, serves as a critical site for a variety of inflammatory processes, including immunity against infections and allergy [9]. Mast cells (MCs) are located within the dermis of the skin [10,11], in close proximity to blood vessels and sensory nerves, constituting about 10% of all dermal immune cells [12]. MCs play an important role in the immune response by detecting external stimuli such as pathogens and toxins; however, dysregulation of their activity or functionality may contribute to hypersensitivity reactions and associated disorders [13,14]. The pathophysiology of CU is not well understood [15,16]; however, MC activation and subsequent degranulation result in the development of urticaria symptoms [17], including wheels and/or angioedema [18]. MC degranulation releases histamine and other mediators such as MC-specific proteases and low levels of cytokines, leading to sensory nerve stimulation, vasodilation, and plasma extravasation as well as to the recruitment of other immune cells to the urticaria site [15]. Abnormal regulation of MC activity may also contribute to AD [19], a chronic inflammatory skin disease which is characterized by recurrent eczematous skin lesions with intense pruritus and type-2 immunity-associated hypersensitivity [20]. It has been observed that the number of MCs in the skin lesions of AD patients are significantly associated with the disease’s severity [21]. The inter-individual differences in MCs among humans may well underlie the heterogeneity of complex inflammatory skin disorders [22]. However, the comprehensive modes of MC activation have not all been unraveled yet.

The activation of MCs occurs (i) via the immunoglobulin E (IgE)-dependent and (ii) IgE-independent pathways [23,24]. It is hypothesized that the highly expressed Mas-related G-protein-coupled receptor X2 (MRGPRX2) constitutes the missing link connecting MCs to AD and CU, at least in selected endotypes where the disorders are non-IgE-dependent [25]. There are many reports on the involvement of IgE-independent reactions, mainly to food additives and drugs, in CU, AD, and other inflammatory disease. However, it is not well understood yet if they act via the MRGPRX2 receptor [26,27,28,29,30,31,32]. This review focuses on MRGPRX2-activated non-IgE MC hypersensitivity, hereby referred to as pseudo-allergic reactions for simplicity [33,34].

## 2. MRGPRX2 Receptor

MRGPRX2, a 37-kDa G-coupled receptor with seven transmembrane domains, is highly expressed in human skin MCs [35]. It is a primate-specific protein with a 53% sequence similarity to its mouse ortholog, MrgprB2 [36,37].

The activation of MRGPRX2 is initiated by many exogenous and endogenous substances, including cationic drugs, neuropeptides, and host defense peptides. These agents are important for mounting antimicrobial defenses and mediating neurogenic inflammation but may also trigger pseudo-allergic reactions [36,38]. The activation of MRGPRX2 induces a signaling cascade leading to elevated cytosolic calcium levels, mediated through Ca++ channels, Gai, Gaq, ERK, PI3K/AKT, and PLCγ, culminating in MC degranulation [13,39]. This process ultimately results in the release of a variety of synthesized mediators [40]. 

It is noteworthy that MRGPRX2 has been shown to be a fundamental signaling protein for MC degranulation in pseudo-allergic reactions, and these reactions themselves may serve as triggering factors in the pathogenesis of conditions such as CU and AD [41].

## 3. Current Insights into MRGPRX2 in CU and AD

**Figure 1:**
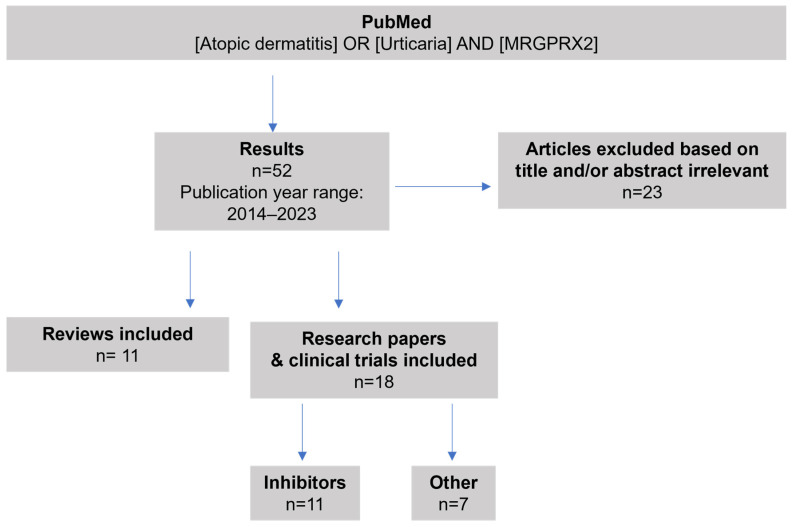
PubMed search strategy and selection of articles for inclusion in this review.

To gain a comprehensive understanding of existing knowledge regarding the role of MRGPRX2 in the context of CU and AD, a thorough literature search was conducted in PubMed utilizing the key words “atopic dermatitis” or “urticaria” and “MRGPRX2”, filtering only for reviews, as described in Figure 1. The findings up until 2023 are summarized in Table 1. 

Previous research has identified and confirmed that there is a role for MRGPRX2 in inflammatory skin diseases. An increased proportion of MRGPRX2-positive MCs and increased levels of this receptor’s expression in MCs have been shown in the skin of CU [19,21,35,42] and AD patients [7,43]. However [15], whether there is really a difference in the MC numbers between patients and healthy controls is not yet certain given that some studies reported increased mast cell numbers in both lesioned and non-lesioned skin samples [44,45]. Other studies found no difference between the mast cell numbers in the skin of CU patients and those in the skin of healthy individuals [46,47,48]. Future studies are needed to resolve this question.

Furthermore, the MRGPRX2 agonist, substance P (SP), also showed elevated serum levels in CU and AD patients that correlated with disease severity [7,35,42,49]. Intradermal administration of SP induced greater wheal reactions in CU patients compared to normal controls, which may be due to increased MRGPRX2 expression in CU patients [21,42]. 

In summary, MRGPRX2 and its ligands are likely involved in the pathogenesis of AD and CU, although the exact dimension of their contribution and mechanisms of action are not known.

**Table 1:** MRGPRX2-related reviews regarding AD and CU.

Disease	Increased Expression in Patients	Increased Ligand Level (SP)	Increase in MCs Number or Activity	Biomarker Potential	Review Source	Original Research Citations
AD	Yes	Yes (plasma)	Yes, number and activity	Yes	[7]	[50]
AD	Not investigated	Not investigated	Yes, number	Not investigated	[43]	[51]
AD	Yes	Yes (skin)	Yes, number	Not investigated	[25]	[50,52,53,54,55,56]
CU	Yes	Not investigated	Yes, number and activity	Not investigated	[57]	[44,45,51,58,59,60]
CU	Yes	Not investigated	Yes, number	Yes	[21]	[51,61]
CU	Yes	Not investigated	Yes, activity	Correlated with disease severity	[42]	[51,62,63,64]
CU	Yes	Not investigated	Not investigated	Not investigated	[19]	[51]
CU, AD	Yes	Yes (in AD, skin)	Yes, number (AD)	Not investigated	[49]	[50,53,65,66,67]
CU, AD	Yes	Yes (serum and skin)	Not investigated	Yes	[35]	[46,51,61,68]
CU, AD	Yes	Not investigated	Not investigated	Not investigated	[69]	[70,71]
Inflammatory skin diseases	Not investigated	Not investigated	Not investigated	Not investigated	[37]	[51]

## 4. Recent Advances in MRGPRX2 Research

To have an overview of current MRGPRX2 research in relation to allergic diseases, we conducted a literature search in PubMed using key words “atopic dermatitis” or “urticaria” and “MRGPRX2”, following the search string as described in Figure 1, however, additionally filtering only for the latest research, from 2020 until 2023. These findings are summarized in Table 2. 

The latest research on the role of MRGPRX2 in skin inflammatory diseases yielded compelling insights into these complex immunological processes. These studies have shown increased MRGPRX2 expression in the MCs of CU patients and in the sera of severe-CU patients [51,62]. Given the central role of MRGPRX2 in pseudo-allergic reactions, research has focused on the discovery of MRGPRX2 activators, which could unravel disease elicitors, inhibitors for pharmaceutical intervention discovery, as well as understanding of the MRGPRX2 signaling pathway which leads to MC degranulation.

### 4.1. MRGPRX2 Activators and Signaling

Further progress has been made regarding MRGPRX2-activating molecules. The high-throughput screening of a library of pharmacologically active compounds has uncovered a class of commonly used drugs that activate MRGPRX2/Mrgprb2, all belonging to the category of cationic amphiphilic drugs [72]. Moreover, new synthetic molecules in the morphine compound family have been shown to specifically activate MRGPRX2/Mrgprb2 [73]. Interestingly, given that more than 90% of AD patients present Staphylococcus aureus colonization in the affected skin areas, recent investigations have shown that the Staphylococcus δ-toxin provokes MC degranulation via MRGPRX2 and that the compound QWF, a MRGPRX2 antagonist, inhibits this activation [74]. Further elucidation of MRGPRX2’s interaction with SP revealed that SP is a balanced MRGPRX2 agonist. Their interaction induces both G-protein-dependent signaling for degranulation and G-protein-independent signaling for β-arrestin recruitment and MRGPRX2 internalization [75]. A highly conserved tyrosine residue within MRGPRX2, Tyr279 plays a crucial role in modulating this response, as the replacement of Tyr^279^ to alanine (Y279A) abolishes SP-induced modulation [75]. β-arrestins have been reported to be negative regulators of MRGPRX2 function in human skin MCs; therefore, strengthening the β-arrestin function could provide novel therapeutic approaches for MRGPRX2-mediated diseases [76,77]. 

Furthermore, in another study, it was described that MRGPRX2 activation led to microphthalmia-associated transcription factor (MITF) phosphorylation and increased MITF levels, while the silencing or inhibition of MITF resulted in decreased MRGPRX2-induced degranulation [78]. Thus, the modulation of MITF and MITF-dependent targets may be considered a therapeutic approach in the context of pseudo-allergic reactions [78].

### 4.2. MRGPRX2 Inhibitors—Potential Therapeutic Candidates

**Figure 2:**
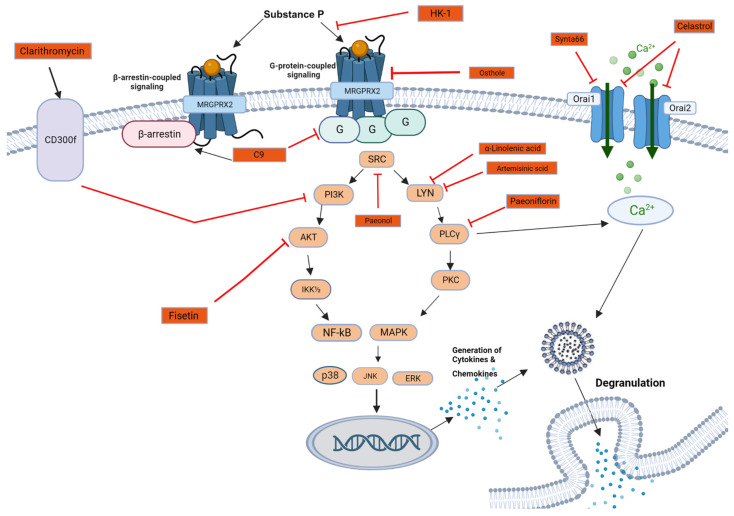
An illustration of the theoretical modes of action of the MRGPRX2 signaling pathway inhibitors. The inhibition symbol represents generalized inhibition including reduction in phosphorylation, reduction in expression, or direct inhibition.

Following the recent discovery of the potential role of MRGPRX2 in pseudo-allergic reactions, there is ongoing vast research towards finding specific inhibitors of the MRGPRX2 signaling pathway for novel therapeutic approaches, and they are summarized in Table 2, while their mechanisms of action are illustrated in Figure 2.

The majority of the compounds currently under investigation have been so far only tested in vitro or in animal models. Among these, C9 stands out as a potent and selective inhibitor of only MRGPRX2-mediated MC degranulation, without affecting Mrgprb2-mediated MC degranulation [79]. Nonetheless, the toxicity and stability of this compound still need to be tested before proceeding with clinical trials. 

Several compounds of plant origin have surfaced as MRGPRX2 inhibitory agents. Celastrol [80], for instance, has exhibited MRGPRX2 inhibition and has been shown to reduce MC production, histamine release, scratching levels, and inflammatory factor expression in mice. The inhibitory effect of Celastrol was reversed upon the overexpression of MRGPRX2 in a mouse model, supporting the notion that this effect may be mediated via MRGPRX2 rather than its orthologs [80]. In very high concentrations, Osthole, an aromatic compound, has displayed inhibitory effects on MGRPRX2 in two in vitro models (LAD2 cells and RBL-2H3 cells steadily expressing MRGPRX2), in MCs isolated from human skin tissue, and in vivo in a mouse model [81]. However, due to the high concentration of the compound needed to achieve said inhibitory effect, Osthole needs to be tested before being labelled as clinically safe. Additionally, the low water solubility of Osthole necessitates modification of the compound. However, it is uncertain whether these modifications preserve its inhibitory function. Two Chinese herb derivatives, Phenol [82] and Paeoniflorin [41], have been reported to decrease CU symptoms. In vitro studies in LAD2 cells and in vivo experiments in mice have confirmed them to be MRGPRX2 inhibitors. 

The rest of the potential inhibitors that have been recently reported in research papers are quite different from one another, which may be due to the fact that the MRGPRX2 signaling pathway is not fully known. An example of this is Synta66, an inhibitor of the Orai channels, i.e., the calcium release-activated calcium channels in murine and human MCs, since, upon MRGPRX2-mediated activation of the inhibitor, an influx of Ca^2+^ is induced [13,83,84,85]. Taking an entirely different direction, the role of Hemokinin-1 (HK-1) in CU has also been investigated, as it was indicated to induce histamine release from LAD2 cells via MRGPRX2 in previous studies [86]. Higher serum levels of HK-1 were detected in healthy controls versus CU patients. Intriguingly, the brief incubation of MCs with HK-1 caused the inhibition of histamine release and did not elicit rapid MRGPRX2 internalization. This suggests that, in healthy controls, HK-1 may regulate and desensitize MRGPRX2-mediated MC activation, thereby preventing MC degranulation by SP [86].

Another approach was to examine an already existing medication. Clarithromycin, a well-known antibiotic, has been revisited for its potential in the treatment of skin inflammatory diseases. It has been shown to inhibit FcεRI- and MRGPRX2-mediated MCs activation in LAD2 cells. Furthermore, in a single-center, self-comparison clinical trial involving 28 CU patients who were not responsive to third-generation antihistamines [84], clarithromycin showed a significant reduction in the wheal and itch symptoms as well as a decrease in the serum cytokine levels (TNF-α, IL-13, IL-4, IL-6, and tryptase) in those patients. Interestingly, CD300f, a leukocyte mono-immunoglobulin-like receptor 3 and an inhibitory immune receptor which is expressed on the surface of neutrophil granulocytes and MCs [87], was shown to be involved in clarithromycin’s inhibitory effect, as the inhibition of said antibiotic decreased significantly after CD300f knockdown in LAD2 cells [84].

Ongoing research is dedicated to identifying specific MRGPRX2 inhibitors for potential therapeutic applications in pseudo-allergic reactions, encompassing diverse compounds. These candidates, which exhibit varying degrees of efficacy in in vitro and animal models, hold promise for addressing MRGPRX2-mediated immune responses in skin inflammatory diseases.

**Table 2:** Latest inhibitors research for potential novel therapeutic approaches.

Inhibitor Name	Disease	Mode of Action/MRGPRX2 Activation Pathway	In Vivo Model	In Vitro Model	Reference
Celastrol	AD	Reduces MC number Inhibits histamine release Suppresses MRGPRX2/ORI Celastrol effect was reversed by overexpression of MRGPRX2	Mouse model	N/A	[80]
Fisetin	CU	MRGPRX2 binding Suppresses calcium mobilization Decreases phosphorylation of Akt, P38, NF-κB, and PLCγ	Mouse model	HEK293, LAD2 cells	[88]
Paeonol	CU	Reduces histamine chemokine release and calcium influx Antagonist of Src kinase activity downstream of MRGPRX2	Mouse model	LAD2 cells	[82]
Artemisinic acid	CU	Not binding directly MRGPRX2 Lyn kinase antagonist Inhibits MC activation	Mouse model	N/A	[89]
C9	CU, AD	Inhibits degranulation, β-arrestin recruitment internalization	Mouse model	RBL-2H3 cells, LAD2 cells, human skin-derived MCs	[79]
Clarithromycin *	CU	Inhibits MC activation Reduces wheal, itch, and serum cytokine levels	Mouse model CU patients (28)	LAD2 cells	[84]
Synta66	CU, AD	Orai channels inhibitor Inhibits SP-induced Ca2+ mobilization, degranulation Inhibits ERK1/2, Akt phosphorylation	Mouse model	LAD2 cells, human skin-derived MCs	[83]
HK-1	CU	Inhibits histamine release Inhibits SP-induced MCs activation	---	Cultured MCs, human skin-derived MCs	[86,90]
α-Linolenic acid	CU	Lyn kinase antagonist Reduces SP-induced MCs degranulation, and histamine and chemokines release	Mouse model	LAD2 cells, HEK293 cells	[90]
Osthole	CU	Does not compete with the MRGPRX2 ligands Reduces MRGPRX2 surface and intracellular expression Inhibits Ca2+ mobilization, degranulation, and cytokine and chemokine production	Mouse model	LAD2 cells, RBL-2H3 cells, human skin-derived MCs	[81]
Paeoniflorin	AD	Inhibits MCs degranulation, histamine release Reduce calcium influx, Downregulates phospho-Erk1/2, P38, PKC, AKT	Mouse model	LAD2 cells	[41]

* In clinical trial.

## 5. Discussion

The outlook of future therapeutic strategies for pseudo-allergic reactions aims to find specific inhibitors targeting MCs, including their activation via the MRGPRX2 receptor [37,49]. However, primarily, it should be confirmed which specific elicitors of MRGPRX2-mediated reactions occur in the context of inflammatory skin diseases such as AD and CU. Therefore, a comprehensive understanding of MRGPRX2’s function and regulation, including the discovery of specific MRGPRX2 regulators, is needed.

According to our findings, there are currently several potential MRGPRX2 inhibitors undergoing investigation. Most studies are in their preliminary stages, when MC-MRGPRX2 activation is prevented due to the addition of the compounds named previously. For some of the inhibitors, there is some (partial) explanation regarding their mechanism of action, whether this it is binding directly to MRGPRX2 (Fisetin [88]) or inhibiting protein activity downstream of MRGPRX2 (Paeoniflorin [41], Paeonol [82], and Synta66 [83]). Most inhibitors in our review were evaluated in in vitro and animal model experiments, and the antibiotic clarithromycin has been clinically tested [84]. Revisiting established antibiotics for the treatment of skin inflammatory diseases is an intriguing approach, as many antibiotics have an additional immunomodulating effect. For example, low-dose doxycycline is used to treat acne and other skin diseases involving an overactive immune system [91]. Some antibiotics, such as dapsone, have been used in the past to treat urticaria [92]. Therefore, it would be worthwhile to explore the old literature regarding the use of various antibiotics in the treatment of CU and AD [93]. Unfortunately, due to the lack of clinical trials meeting modern standards, these treatments have often been overlooked or classified in treatment guidelines as having an insufficient level of evidence [94]. Notably, antibiotics, especially those for *H. pylori* and dapsone, have demonstrated improved remission rates and symptom relief in CU cases, with few adverse events, warranting further studies [93].

One of the limitations in MRGPRX2 research is the lack of animal models for studying these primate-specific receptors [95]. Most in vivo research was conducted in the mouse model C57BL/6, using Mrgprb2, the ortholog of MRGPRX2. The work by Bawazir et al. [79] has raised a question regarding the applicability of MRGPRX2 inhibitor study results from mice, since the mouse ortholog has other variants with a lot of sequence similarities, potentially leading to cross-activation. These variants differ from MRGPRX2, challenging the direct translation of animal model-based findings to human conditions [79]. Nonetheless, to address this challenge, mouse models generating MRGPRX2-expressing MCs have been developed [96]. 

In reviewing the presented inhibitors, we highlight three molecules that show the highest therapeutic potential so far. Bawazir et al. [79] presented C9 as a potent and, importantly, selective inhibitor of only MRGPRX2-mediated MC degranulation. Yao C et al. [80] found that the improvements of AD caused by Celastrol were reversed by means of treatment with MRGPRX2 overexpression, indicating that Celastrol might affect AD via MRGPRX2. Clarithromycin already showed positive outcomes in a preliminary clinical trial in the treatment of CU. Future studies on these compounds are needed to elucidate their clinical significance.

## 6. Conclusions

In summary, the identification and characterization of MRGPRX2 have ushered in a significant paradigm shift in our understanding of mast cell (MC) biology, shedding light on non-IgE-mediated clinical manifestations which are mediated by MCs [25]. The emergence of high-affinity receptor inhibitors targeting MRGPRX2 presents a promising and innovative therapeutic approach for addressing MC-mediated diseases including CU and AD. Future studies encompassing clinical trials will be needed to verify the clinical efficacy and safety profiles of these inhibitors. Consequently, additional research endeavors are warranted to explore the potential therapeutic applications of these inhibitors not only in CU and AD but also in a broader spectrum of pseudo-allergic reactions.

## Data Availability

No new data were created.

## Abbreviations

MCs	Mast cells
CU	Chronic urticaria
AD	Atopic dermatitis
IgE	Immunoglobulin E
MRGPRX2	Mas-related G-protein-coupled receptor X2
